# Comparing Electrochemical Performance of Thin-Film Ti-Pt Microelectrodes on Planar and Non-Planar Glass Substrates for Lab-on-a-Chip Applications

**DOI:** 10.3390/mi17030318

**Published:** 2026-03-04

**Authors:** Karolina Kołczyk-Siedlecka, Zbigniew Szklarz, Elizaveta Vereshchagina, Aina Herbjørnrød, Paul Wittendorp, Shruti Jain, Piotr Żabiński, Aldona Garbacz-Klempka, Paweł Wójcik

**Affiliations:** 1 Faculty of Non-Ferrous Metals, AGH University of Krakow, 30-059 Kraków, Poland; 2 Department of Microfluidic Electrochemistry, Redoxme AB (redox.me), 30-054 Kraków, Poland; 3 Faculty of Foundry Engineering, AGH University of Krakow, 30-059 Kraków, Poland; 4 Department of Smart Sensors and Microsystems, SINTEF Digital, 0373 Oslo, Norway; elizaveta.vereshchagina@sintef.no (E.V.);; 5 Research & Development Department, Redoxme AB (redox.me), 602 33 Norrköping, Sweden

**Keywords:** thin-film microelectrodes, electrochemical characterization, lab-on-a-chip systems

## Abstract

Miniaturization plays a critical role in modern microsystem engineering by enabling automation, reducing material consumption, and lowering operational costs. In electrochemical microsystems, thin-film electrodes are widely adopted due to their favorable surface-to-volume ratio and compatibility with scalable microfabrication processes. This study investigates the electrochemical performance, reliability, and reproducibility of titanium–platinum (Ti-Pt) thin-film microelectrodes patterned on both planar and non-planar glass substrates. The electrodes were fabricated using standard photolithography and physical vapor deposition (PVD) techniques, with curvature variation introduced to assess the effect of substrate topology on functional properties. Electrochemical characterization was conducted using classical cyclic voltammetry and impedance spectroscopy, with performance benchmarked against conventional platinum electrodes. The results show that Ti-Pt electrodes exhibit stable and repeatable behavior regardless of substrate curvature, with minor variations in impedance characteristics. These findings confirm the suitability of Ti-Pt microelectrodes for integration into complex lab-on-a-chip architectures, including those involving non-planar geometries.

## 1. Introduction

Electrochemistry is a subfield of physical chemistry dealing with the study of chemical processes [1]. Electrochemistry is a versatile discipline with a wide range of applications and analytical capabilities. Its continued development and integration into various fields promise innovative solutions to complex challenges and deeper insights into chemical processes, materials [2,3,4,5], and biological systems [6,7]. It has found applications across various fields due to its versatility and ability to provide valuable insights into chemical reactions and material properties. For example, electrochemistry plays a central role in batteries [8,9,10,11,12,13] and fuel cells [14], catalysts [15], enabling energy storage [16] and conversion for many applications, from portable electronics to electric vehicles [17] and more [18,19,20,21,22].

Electroanalytical techniques are fundamental tools in analytical chemistry [23]. They are used for quantitative analysis, identifying substances, and determining reaction kinetics. Electrochemical methods offer precise control over reaction conditions, making them suitable for studying reaction kinetics, thermodynamics, electroactive species’ behavior, and redox reactions in biological systems, aiding in the development, for example, of novel drugs and diagnostic tools [24,25]. Electrochemical sensors, individually or in combination with other sensing methods, are also widely used for environmental monitoring, food analysis, and more. They can be used in the detection of pesticides, heavy metals, and gases in air and water, contributing to environmental protection and public health.

Lab on Chip (LoC), also referred to as micro total analysis systems, represents a groundbreaking innovation in the field of analytical chemistry, biology and more [26,27]. Miniaturization has revolutionized the way experiments and analysis can be conducted, making it possible to perform complex laboratory procedures on a chip-sized platform. This miniaturization allows researchers to perform various experiments with small sample volumes, reduce reagents consumption, and obtain faster results. LoC technology can be cost-effective over time, making advanced analytical techniques more accessible [28,29].

LoC has found applications across numerous fields, including various types of sensors [30,31,32,33,34], the diagnosis of pathogens [35,36,37,38], clinical diagnostics [39,40,41], environmental monitoring [42], chemical and drug monitoring [43,44,45], and more [46,47,48,49,50]. The controlled flow of fluids within microchannels on LoC platforms enables precise manipulation of samples and reagents. Automation is simplified, reducing the risk of human error, and increasing experimental reproducibility. LoC devices often incorporate various sensors, such as electrochemical [51], optical [52], and biological sensors, enhancing their analytical capabilities. This integration often simplifies real-time monitoring and data acquisition.

In the realm of modern science and technology, the integration of miniaturized electrochemical sensors into microfluidic devices has emerged as a promising avenue for various applications, ranging from chemical analysis to biomedical diagnostics. Among the essential components of the microfluidic platforms are the thin-film electrode materials [52], which play a significant role in facilitating electrochemical processes [53,54]. The reliability of thin-film electrodes in miniaturized electrochemical flow cells remains a major limiting factor for applications requiring a long lifetime and reproducibility in analyses. Integrating electrodes into microchannels introduces additional challenges, e.g., electrodes breaking over the channel walls. This study advances the state of the art by benchmarking electrode reliability patterned on both planar and curved microfluidic geometries, enabling a general comparison of how surface topology influences stability and electrochemical performance. Although spray-coating methods are established [55,56], they are not yet routinely employed for miniaturized electrochemical systems integrated with microfluidics, as it is hard to meet the requirements necessary for reliable and prolonged electrochemical analysis. This paper provides foundational groundwork for challenges in the fabrication and performance of electrodes patterned over non-conformal substrates by presenting a proof-of-concept, while not pursuing extensive optimization of the spray-coating parameters or studies on the yield of uniform channel coverage.

In this context, platinum (Pt) microelectrodes have garnered considerable attention due to their exceptional electrochemical properties. In this paper, the influence of microelectrode geometry and morphology on electrochemical performance was determined. Pt, known for its catalytic activity and stability, has demonstrated remarkable potential as an electrode material, particularly when scaled down to microscale dimensions. Moreover, the successful deposition of Pt microelectrodes inside the micro channels etched inside the glass substrates represents a significant advancement in the field of microfluidics. The microchannel Pt electrodes may offer a unique opportunity to precisely control and manipulate chemical reactions within microfluidic channels, making them indispensable for applications requiring high sensitivity and precision. Comparative studies were performed on thin-film electrodes and classical microelectrodes in the form of a wire embedded in glass in order to determine the quality of the obtained coatings and their usefulness in the performance of precise electrochemical measurements.

Ferricyanide/ferrocyanide is a commonly used redox couple for measuring the mass transfer performance of electrochemical reactors; it is applied as an electrochemical standard to characterize the interfacial processes of new electrode materials [57,58,59].

This paper aims to delve into the electrochemical properties of Pt microelectrodes deposited on glass substrates and their implications for the future of microfluidic chip technology. Through comprehensive experimentation and analysis, the factors influencing the electrochemical behavior of these microelectrodes, such as electrode size, surface morphology, and shape of substrate, were tested. This research will allow us to take the next step in the production of electrochemical sensors with the highest possible precision of measurements.

## 2. Materials and Methods

**Figure 1:**
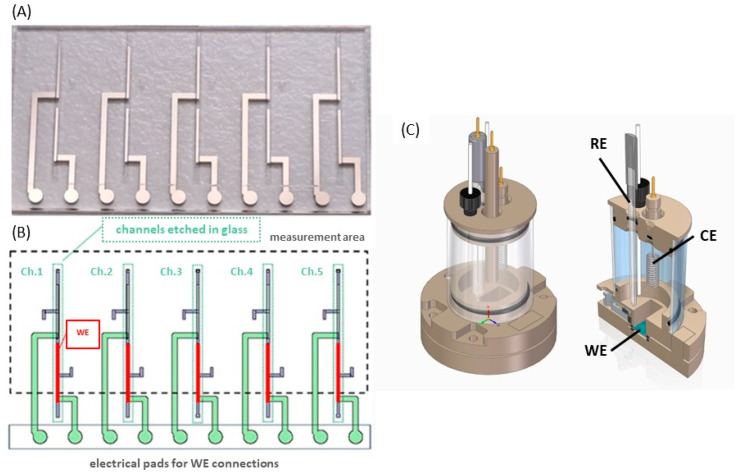
(**A**) Picture of used planar chip, (**B**) design of Ti-Pt microelectrodes, (**C**) the electrochemical cells used in measurements.

Figure 1A shows a picture of a glass chip with five parallel measurement areas. In Figure 1B, the schematic drawing is posted to show the details of the chip. The black dashed line shows the area exposed to the electrolyte. The green dotted lines indicate the area of the channels inside which the electrodes are located. The active area of the microelectrodes which were used throughout the study is shown in red. They are characterized by a smaller surface area, and in the case of a chip with channels, the Pt electrode is entirely located inside the channel, which allows for a better comparison of the type of glass substrate. The surface area of this part equals 0.32 mm^2^ and it was fully immersed in electrolyte during measurements. All electrochemical measurements were performed on the same electrode design. The electrodes were patterned on planar and non-planar (with microchannels) substrates, which were cut from 150 mm diameter double-side polished glass wafers (PlanOptik, Elsoff, Germany).

Both planar (without microchannels) and non-planar (with microchannels) wafers went through the same process for the deposition of thin-film Ti-Pt electrodes. The wafers were rinsed in the solution of ammonium hydroxide for about 10 min, following a rinse in DI water. A total of 100 nm Pt with a 12 nm Ti adhesion layer was deposited using an AMAT Endura cluster tool. In the case of wafers with channels, the patterned side was coated with metal. The equipment used for spray-coating was an EVG^®^101 Advanced Resist Processing System (Sankt Florian am Inn, Austria). The wafers with microchannels relied on a lithography process with a spray-coat resist (AllResist, Strausberg, Germany), while the planar wafers used a spin-coating process with a 2.6 µm thick positive photoresist (HIPR 6512, Fujifilm, Tokyo, Japan); the wafers were processed using an EVG^®^101 Advanced Resist Processing System (Sankt Florian am Inn, Austria). Both types of wafers were exposed using the MA150 mask aligner, followed by development in a dedicated developer, inspection, and hard bake. The etching of Ti-Pt for both wafer types was carried out in a parallel-plate etcher PlasmaPro 80 RIE (Oxford Instruments, Abingdon, UK). After this, the resist was removed from the Tepla 300 plasma ashing system. Further, the wafers were inspected in an optical microscope and diced using dicing saw Disco DAD321. The resulting chips had dimensions of ca. 13.85 × 25.5 mm^2^.

Next, the electrochemical measurements were carried out in 0.1 M KCl water solution or 1 mM hexacyanoferrate (II)/(III), where 0.1 M KCl was used as a supporting electrolyte by using the dedicated electrochemical cell (Figure 1C), designed and manufactured by Redoxme AB (Department of Microfluidic Electrochemistry, Krakow, Poland). The cells were made of polyether ether ketone (PEEK), which is characterized by high chemical and mechanical resistances. This special vessel ensures the fixed positioning of electrodes in every measurement. For each electrochemical test, it was filled with the same volume of electrolyte—20 mL—and was tightly closed, limiting the access of the air. The experiments were run at room temperature. All reagents were characterized by analytical purity (WARCHEM sp. z o.o., Zakręt, Poland).

The measurements collected from thin-film electrodes were compared with wire-based Pt microelectrodes (Redoxme AB, Norrköping, Sweden) with 100 µm diameter placed in a glass tube. Wire-based microelectrodes are commonly used in electrochemistry, which is why such a comparison was made. For all tests, either Pt wire or a thin-film Ti-Pt microelectrode acted as a working electrode (WE), the platinum spiral wire was used as a counter electrode (CE) and the Ag/AgCl electrode was used as a reference electrode (RE).

A series of electrochemical measurements were performed using the cyclic voltammetry (CV) method with different scan rates in the range from 10 to 100 mV/s. For each scanning rate, 30 CV scans were performed. Then, 1000 CV scans with a scan rate of 100 mV/s were taken for long-term electrochemical stability measurements. All tests were performed in the potential range from −0.25 to 1.25 V vs. Ag/AgCl to include hydrogen and oxygen evolution processes. In the case of measurements carried out in hexacyanoferrate (II)/(III) solution, 10 CV scans were performed at different scan rates from 10 to 200 mV/s in the potential range of ±0.1 V vs. OCP (Open Circuit Potential). Then, 10 CV curves were determined with a scan rate of 100 mV/s with potential ranges from ±0.3 to ±1.2 V vs. OCP.

In the case of CV curves, the average current density and standard deviation values in characteristic peaks were determined. Such calculations make it possible to define the repeatability of the results obtained on individual electrodes and compare them to each other.

Electrochemical measurements were performed using the MULTI AUTOLAB M204 (Utrecht, The Netherlands) galvanostat–potentiostat equipped with an electrochemical impedance spectroscopy module and the NOVA 2.1.5 software. The microfabricated Ti-Pt electrodes were analyzed using an optical/confocal microscopy (Nikon Eclipse L100, Tokyo, Japan), a scanning electron microscopy (JEOL Microscope, Tokyo, Japan) and an atomic force spectroscopy (NtegraAura microscope, NT MDT, Moscow, Russia).

## 3. Results and Discussion

**Figure 2:**
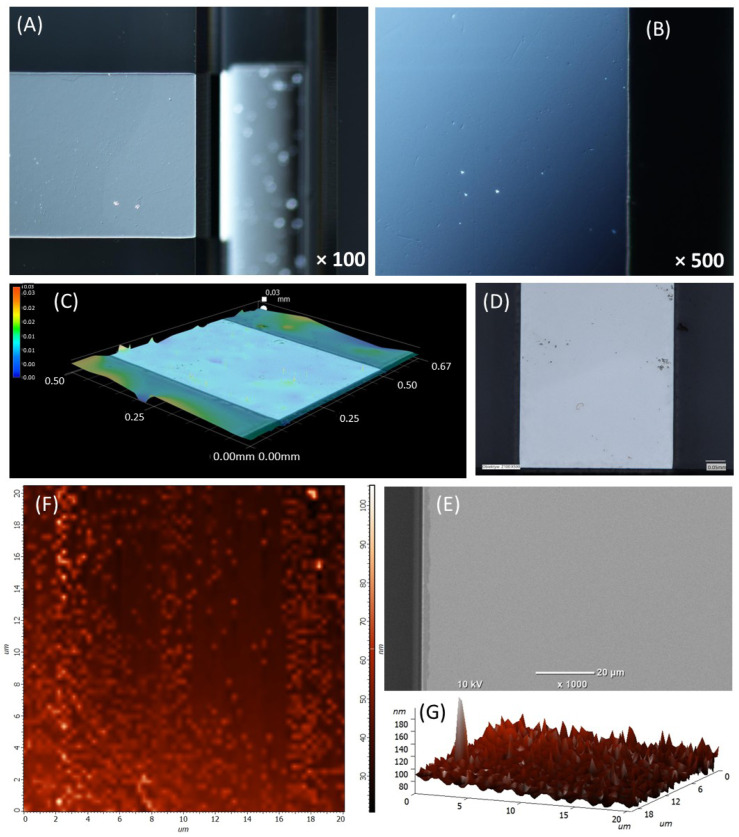
Surface analysis of Pt microelectrode on planar chip. (**A**,**B**) Optic microscopy pictures, (**C**,**D**) confocal microscopy pictures, (**E**) SEM picture (**F**,**G**) AFM analysis.

The analysis of the surface of thin-film Ti-Pt microelectrodes on a planar substrate is presented in Figure 2. Optical microscope images (Figure 2A,B) with different magnifications show that the metal film surface is smooth. This was also confirmed by confocal microscopy measurements (Figure 2C,D); there were no discontinuities observed in the coatings. Only slight irregularities in the form of dots were observed, which are probably related to contamination from the environment. A picture from a scanning microscope with a magnification of 1000× (Figure 2E) shows that the edges of the electrode are sharp and adhere well to the substrate. AFM images (Figure 2F,G) show that the coating is homogeneous. The average roughness Ra determined in the area of 20 µm × 20 µm is equal to 12.3661 nm.

**Figure 3:**
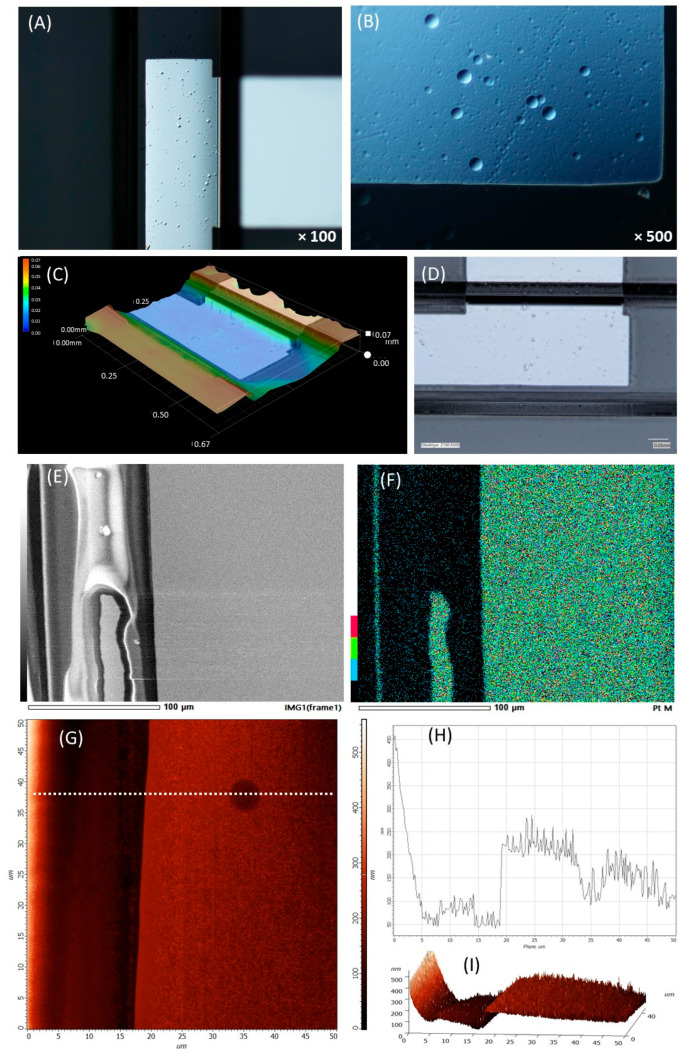
Surface analysis of Pt electrode in channel. (**A**,**B**) Optic microscopy pictures, (**C**,**D**) SEM picture and Pt mapping analysis, (**E**,**F**) confocal microscopy pictures, (**G**–**I**) AFM analysis.

The case of the Pt electrodes on a non-planar substrate, i.e., located inside the microfluidic channels, is presented in Figure 3. The coating is characterized by some unevenness with dot-like heterogeneities, which is visible under the optical microscope (Figure 3A). By observing the shadow on the sample (Figure 3B), it can be seen that these are spherical holes. These defects were probably formed during the etching of the glass, which is a main step in the production of channels in this type of substrate. Despite the non-uniformity of the glass channel surface, the thin metal film is continuous. Analysis using a confocal microscope (Figure 3C,D) confirmed whether there were any discontinuities in metal coating observed, especially on the wall of the channel. During the inspection, particular attention was paid to the walls and bottom of the channel. The SEM picture (Figure 3E) and Pt mapping analysis (Figure 3F) of the area close to the edge of the electrode inside the channel show that, in addition to the Pt electrode, additional areas inside the channel were also deposited due to the specification of the metal deposition process. However, these are areas that are not connected with the electrode, so they should not disturb the operation of the chip. The edges of the electrode appear sharp and adhere well to the substrate. During the atomic force microscopy (Figure 3G) analysis, characteristic circular areas were observed on the surface of Ti-Pt deposited inside the channel. Analysis of the profile (Figure 3H) along the line marked in the figure (Figure 3G) allows us to conclude that these areas are spherical holes. They may have appeared during the etching of the glass substrate in 50% HF, which is a key step of the process of channel preparation. The diameter of such a hole is approx. 10 µm. The 3D image (Figure 3I) presents the area with the channel wall and the edge of the Pt electrode is visible; it is clearly defined. The average roughness determined in the area of 20 × 20 µm without any surface defects is equal to 30.0018 nm.

**Figure 4:**
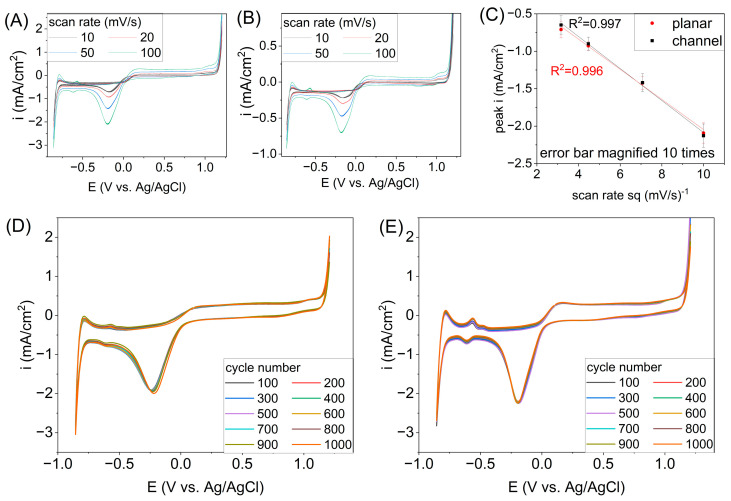
Effect of scan rates on the electrochemical response in 0.1 M KCl registered for the Pt microelectrodes on planar chip (**A**) and chip with channels (**B**). Linear fitting of peak current density −0.18 V vs. Ag/AgCl depending on scan rate (**C**). Long-term measurements on planar chip (**D**) and chip with channels (**E**).

Figure 4 presents the results of cyclovoltammetric measurements in 0.1 M KCl electrolyte. The graph marked (Figure 4A) shows the results obtained for a Pt electrode on a planar substrate. Graph (Figure 4B) shows the results of analogous measurements, but they were made on a substrate with channels.

In analyzed variants, CV curves typical for platinum electrodes in the KCl environment were recorded, including a peak near the potential −0.18 V vs. Ag/AgCl. In the case of the substrate geometry, a certain influence is observed not on the shape of the curve itself, but on the current density in the characteristic peak. The linear fit is very good; in both cases, R^2^ is 0.997 or 0.996 (Figure 4C). Measurement error bars have been multiplied 10 times to obtain better visibility in the graph. In the case of long-term measurements consisting of 1000 CV measurements (Figure 4D,E), some differences in the course of the curves were observed, which may result from differences in surface morphology between the planar and inside the channel electrodes. For the planar electrode (Figure 4D), the average current density for the peak was equal to −1.880 mA/cm^2^, and the standard deviation was equal to 2.323%. In the case of the chip with channels (Figure 4E), the average current density for the peak was equal to −2.227 mA/cm^2^, and the standard deviation was equal to 0.80%. This behavior may be due to the fact that these measurements are long-term and, in the case of measurements at the planar electrode, some random perturbations may have occurred.

Table 1 presents the analysis of the anodic peak intensity and standard deviation values depending on the scan rate and electrode type. The values were obtained during measurements in 0.1 M KCl electrolyte; CV curves are shown in Figure 4. Standard deviations will allow for potential use as an electrochemical sensor. The type and size of the electrode are not affected; the higher the scan rate, the smaller the measurement error. When measurements at the lowest scan rate of 10 mV/s were performed, the average standard deviation was less than 2%. However, the standard deviation values recorded for the various scan rate values were averaged for each analyzed electrode. Thus, in the case of electrodes on a planar surface, the average standard deviation value is equal to 0.87%. A chip with channels is characterized by larger standard deviation value and it is equal to 1.11%. However, in this case, these values are still satisfactory, taking into account the fact that reactions were tested in a wide range of potentials. Such differences, depending on the surface geometry, may result from the fact that the metallic layer has different morphology, including roughness. In the case of a metallic layer deposited on a flat glass substrate, where the roughness coefficient was equal to 12.3661 nm, the standard deviations values are lower. The higher roughness of the metallic layer (30.0018 nm) deposited inside the channel results in larger standard deviations of the analyzed peaks.

**Figure 5:**
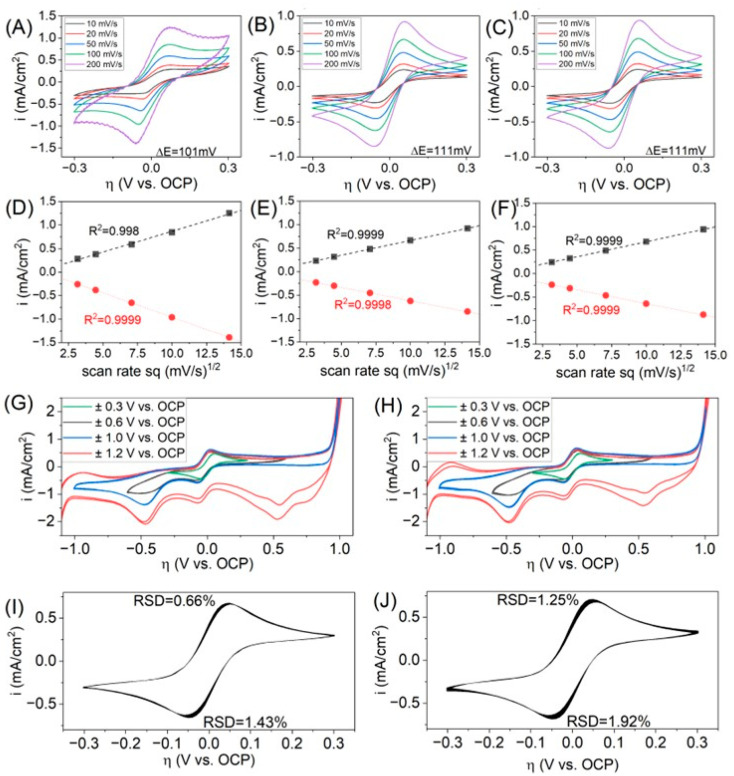
The dependence of CV curves on scanning speed and peak fitting for the Pt wire-based microelectrode 100 µm Pt microelectrode (**A**,**D**), Pt thin-film planar microelectrode (**B**,**E**), and Pt thin-film microelectrode inside the channel (**C**,**F**). CV measurements for wider potential ranges on planar chip (**G**) and chip with channels (**H**). Long-term measurements on planar chip (**I**) and chip with channels (**J**). Used electrolyte: 1 mM potassium hexacyanoferrate (II)/(III) in 0.1 M KCl.

Several measurement series were performed in a solution of 1 mM potassium ferricyanide/ferrocyanide K_3_[Fe(CN)_6_]/K_4_[Fe(CN)_6_]; the supporting electrolyte was 0.1 M KCl solution. The results are presented in Figure 5. The electrochemical reaction (1) is often taken as a simple and fast one-electron transfer at the outer spheres of each complex [60,61,62,63]:

(1) $${{\left[Fe{\left(CN\right)}_{6}\right]}^{3-}}_{\left(aq\right)}+\;e^{-}\;⇄{{\;[Fe{\left(CN\right)}_{6}]}^{4-}}_{\left(aq\right)}$$

First, 10 CV scans are presented in Figure 5A–C. The maximum of the anodic and cathodic peaks, the values of the potential difference ΔE and the linear relationship depending on the square root of the scan rate were determined (Figure 5D–F). Subsequently, 10 CV curves were determined with a scanning speed of 100 mV/s with wider potential ranges. For the clarity of the graphs (Figure 5G,H), scans number 2 and 10 are presented. Then, after emptying the cells and refilling with fresh electrolyte, long-term tests of 1000 CV were performed in the potential range of ±0.1 V vs. OCP for a scan rate of 100 mV/s (Figure 5I,J).

Table 2 presents the OCP values for CV measurements performed in 1 mM hexacyanoferrate (II)/(III) solution with 0.1 M KCl as a supporting electrolyte with various scan rates, presented in Figure 5A–C. Before each CV scan, OCP was recorded. It is clearly visible that, in the case of a Pt wire-based microelectrode embedded in glass, the OCP values are different from printed electrodes. Moreover, depending on the scan rate, the OCP values change; in the case of the wire-based microelectrode, the standard deviation value of OCP is greater than 5%. In comparison, thin-film electrodes have standard deviation values below 2%. This suggests a greater electrochemical stability of the systems in which the electrodes are printed. This may be due to the presence of reaction products on the surface of the classic microelectrode with a diameter of 100 µm.

Figure 5A–C present CV curves for different scan rate values in a regular potential range, i.e., η ± 0.3 V vs. OCP, where the η value is overpotential, defined as the difference in the operating potential and the equilibrium potential. Regardless of the type of Pt electrode, typical and well-known reduction/oxidation peaks suitable for reaction (1) are observed. However, in the case of the Pt wire-based microelectrode (A), at higher scan rate values, deflections from the curves are observed at extreme potentials, which may indicate some additional interfering reactions, which may disturb the measurements of the redox reaction. Moreover, at a scan rate of 200 mV/s, deviations were observed in the CV curve. During the measurements on thin-film electrodes, the same curves (B, C) were obtained, which is logical, since the dimensions of the electrodes are equal. The only difference between the systems is the substrate shape (planar vs. non-planar). The differences in the shape of CV curves are certainly caused by different surface geometries. To some extent, it is observed in the graphs of the dependence of the current density in the anodic and cathodic peaks depending on the square root of the scan rate value (D–F). In most cases, the coefficient of determination R^2^ is equal to 0.9999, but in the case of the cathode peak recorded for wire-based microelectrodes, the R^2^ value is equal to 0.998, which differs significantly from the other recorded values.

In subsequent measurements (Figure 5G,H), CV curves were obtained in extended potential ranges to η ± 1.2 V vs. OCP that include irreversible reactions, such as the adsorption of iron ions or hydrogen/oxygen evolution. A detailed identification of peaks and a description of the processes occurring during analogous measurements were reported previously [64].

After the measurements, the electrolyte was replaced and long-term measurements (Figure 5I,J) were carried out consisting of 1000 CV scans at a rate of 100 mV/s for the potential range η ± 0.3 V vs. OCP. The curves, as in the previous cases, are characterized by typical anodic and cathodic peaks. Relative standard deviations were determined from the recorded peak maxima and, in this case, some differences were observed. Namely, in the case of the thin-film electrode on the planar substrate, the RSD values for the cathodic and anodic peaks are equal to 0.66 and 1.43%, respectively, while in the case of the Pt electrode embedded inside the channel, these values are equal to 1.25 and 1.92%. As in the case of measurements performed in 0.1 M KCl (Table 1), larger RSD values are observed in the case of the electrode embedded inside the channel.

As before, in the 0.1 M KCl electrolyte, a statistical measurement of the recorded peaks was performed in the ferrocyanide electrolyte (Table 3). The data in Table 3 refer to the CV graphs shown in Figure 5A–C. In this case, the current densities of the cathodic and anodic peaks assigned to reaction (1) were analyzed. Three types of electrodes were compared—a wire-based Pt electrode and thin-film electrodes on a planar glass substrate and inside the channel. There is an observed tendency that, in the case of large scan rate values, standard deviations are generally smaller than in the case of lower scan rate values. The exceptions are measurements for a scan rate of 200 mV/s carried out with wire-based electrodes—the standard deviations are clearly larger. This is probably due to the clearly different course of the curves (Figure 5A). The peaks recorded on the wire-based microelectrode are characterized by larger values of the average standard deviation compared with thin-film Pt electrodes. This indicates better electrochemical stability and better repeatability of CV measurements.

## 4. Conclusions

In this study, the performance of the thin-film Ti-Pt has been described. Electrochemical tests were carried out in a three-electrode system, where the working electrodes were microelectrodes patterned on planar and non-planar glass substrates. The electrodes were tested in typical electrolytes used in research work, i.e., 0.1 M KCl and solutions of 1 mM hexacyanoferrate with supporting electrolyte 0.1 M KCl. Differences in Pt thin-film microelectrodes were characterized mainly by surface quality and roughness, which resulted from the type of substrate—smooth or etched glass. Measurement errors were determined based on the standard deviation of the current density of peaks characteristic of the obtained CV curves.

CV measurements with different scan rate values showed that Pt electrodes patterned in the channels have a slightly larger measurement error, but it is still a satisfactory value. In the case of measurements in 0.1 M KCl electrolyte, the average standard deviation for planar Pt microelectrodes was below 1%, in channels—above 1%. However, during measurements in a 1 mM hexacyanoferrate solution, the RSD values below 0.3% were obtained. Measurements carried out with this electrolyte were compared with a wire-based Pt microelectrode. Larger measurement errors were obtained in the case of using a classical wire-based microelectrode, and it was possible to obtain satisfactory results in a smaller range of scan rate values. The obtained comparative results allow us to conclude that the obtained thin-film electrodes have a potentially wider application and it is possible to perform more precise electrochemical measurements.

The main conclusion of this work is the fact that thin-film Pt microelectrodes can be successfully used in electrochemical measurements where high accuracy is required. Moreover, studies have shown that the accuracy of measurements is better than when using classic wire-based microelectrodes. The fabrication method described for patterning thin-film electrodes on non-planar surfaces is suitable for implementation in LoC devices across a wide range of dimensions.

The research work carried out and presented in this paper is a preliminary stage of the development of the production of microsystems for electrochemical sensors. The conclusions from this experimental work will allow for the development of microelectrodes made of other metals and alloys, which will be characterized by high repeatability and accuracy.

## Figures and Tables

**Table 1:** Average density calculation of peak −0.18 V vs. Ag/AgCl analysis for different thin-film Pt microelectrodes, depending on the scan rate in the 0.1 M KCl electrolyte.

Thin-Film Pt Electrode Type	-	Scan Rate (mV/s)				Average RSD (%)
		100	50	20	10	-
Planar	i (mA/cm^2^)	−2.092	−1.425	−0.928	−0.710	0.87
	Std. dev (%)	0.65	0.63	0.61	1.58	
Channel	i (mA/cm^2^)	−2.128	−1.419	−0.900	−0.649	1.11
	Std. dev (%)	0.73	0.83	0.98	1.90	

**Table 2:** OCP analysis for various Pt electrode types in 1 mM potassium hexacyanoferrate (II)/(III) in 0.1 M KCl electrolyte.

Scan Rate (mV/s)	Electrode Type		
	100 µm Pt	Pt/Planar	Pt/Channel
10	0.302	0.221	0.220
20	0.28	0.231	0.228
50	0.279	0.229	0.228
100	0.26	0.226	0.225
RSD [%]	5.31	1.66	1.45

**Table 3:** Average density calculation of anodic and cathodic peak analysis for different Pt microelectrodes, depending on scan rate in 1 mM ferrocyanide in 0.1 M KCl electrolyte, i_c_—current density of cathodic peak, i_a_—current density of anodic peak.

Electrode Type		Scan Rate (mV/s)					Average RSD (%)
		200	100	50	20	10	
100 µm Pt	i_c_ (mA/cm^2^)	1.246	0.853	0.594	0.433	0.359	0.40
	Std. dev (%)	0.42	0.14	0.31	0.71	0.39	
100 µm Pt	i_a_ (mA/cm^2^)	−1.398	−0.965	−0.654	−0.384	−0.310	0.28
	Std. dev (%)	0.76	0.05	0.05	0.06	0.50	
Pt planar	i_c_ (mA/cm^2^)	0.920	0.667	0.482	0.317	0.236	0.21
	Std. dev (%)	0.10	0.13	0.31	0.24	0.28	
Pt planar	i_a_ (mA/cm^2^)	−0.851	−0.625	−0.456	−0.305	−0.231	0.13
	Std. dev (%)	0.05	0.09	0.20	0.17	0.12	
Pt channel	i_c_ (mA/cm^2^)	0.938	0.679	0.491	0.324	0.242	0.27
	Std. dev (%)	0.19	0.12	0.36	0.31	0.35	
Pt channel	i_a_ (mA/cm^2^)	−0.876	−0.644	−0.469	−0.313	−0.239	0.18
	Std. dev (%)	0.16	0.11	0.25	0.19	0.18	

## Data Availability

Data will be made available on request.

## Abbreviations

The following abbreviations are used in this manuscript:

PVD	Physical Vapor Deposition
LoC	Lab on Chip
PEEK	Polyether Ether Ketone
WE	Working Electrode
CE	Counter Electrode
RE	Reference Electrode
CV	Cyclic Voltammetry
OCP	Open Circuit Potential
AFM	Atomic Force Spectroscopy
SEM	Scanning Electron Microscopy
RSD	Relative Standard Deviation
